# Emotion regulation in disordered eating: Psychometric properties of the Difficulties in Emotion Regulation Scale among Spanish adults and its interrelations with personality and clinical severity

**DOI:** 10.3389/fpsyg.2015.00907

**Published:** 2015-06-30

**Authors:** Ines Wolz, Zaida Agüera, Roser Granero, Susana Jiménez-Murcia, Kim L. Gratz, José M. Menchón, Fernando Fernández-Aranda

**Affiliations:** ^1^Department of Psychiatry, University Hospital of Bellvitge-IDIBELLBarcelona, Spain; ^2^Ciber Fisiopatologia Obesidad y Nutrición (CIBEROBN), Instituto Salud Carlos IIIBarcelona, Spain; ^3^Department of Clinical Sciences, School of Medicine, University of BarcelonaBarcelona, Spain; ^4^Department of Psychobiology and Methodology, University Autònoma of BarcelonaBarcelona, Spain; ^5^Department of Psychiatry and Human Behavior, University of Mississippi Medical CenterJackson, MS, USA; ^6^Ciber Salud Mental (CIBERSAM), Instituto Salud Carlos IIIBarcelona, Spain

**Keywords:** eating disorder, emotion regulation, difficulties in emotion regulation scale, personality types, harm avoidance, self-directedness, vulnerability to psychopathology

## Abstract

**Objective:** The aims of the study were to (1) validate the Difficulties in Emotion Regulation Scale (DERS) in a sample of Spanish adults with and without eating disorders, and (2) explore the role of emotion regulation difficulties in eating disorders (ED), including its mediating role in the relation between key personality traits and ED severity.

**Methods:** One hundred and thirty four patients (121 female, mean age = 29 years) with anorexia nervosa (*n* = 30), bulimia nervosa (*n* = 54), binge eating (*n* = 20), or Other Specified Feeding or Eating Disorders (*n* = 30) and 74 healthy control participants (51 female, mean age = 21 years) reported on general psychopathology, ED severity, personality traits and difficulties in emotion regulation. Exploratory and confirmatory factor analyses were conducted to examine the psychometrics of the DERS in this Spanish sample (Aim 1). Additionally, to examine the role of emotion regulation difficulties in ED (Aim 2), differences in emotion regulation difficulties across eating disorder subgroups were examined and structural equation modeling was used to explore the interrelations among emotion regulation, personality traits, and eating disorder severity.

**Results:** Results support the validity and reliability of the DERS within this Spanish adult sample and suggest that this measure has a similar factor structure in this sample as in the original sample. Moreover, emotion regulation difficulties were found to differ as a function of eating disorder subtype and to mediate the relation between two specific personality traits (i.e., high harm avoidance and low self-directedness) and ED severity.

**Conclusions:** Personality traits of high harm avoidance and low self-directedness may increase vulnerability to ED pathology indirectly, through emotion regulation difficulties.

## Introduction

Difficulties in emotion regulation have been identified as a transdiagnostic risk factor for the development and maintenance of numerous forms of psychopathology (Aldao et al., 2010; Hechtman et al., 2013), including eating disorders (ED; Svaldi et al., 2012). In male and female student populations, emotion regulation abilities are related to disordered eating and body-dissatisfaction (Lavender and Anderson, 2010; Ambwani et al., 2014; Cooper et al., 2014). Moreover, literature suggests that inhibited or disinhibited food intake and, thus, the development of an ED, may function to regulate emotions in the absence of more adaptive emotion regulation strategies (Macht, 2008; Fox and Power, 2009; Haynos and Fruzzetti, 2011; Brockmeyer et al., 2012; Naumann et al., 2014; Leehr et al., 2015). This is supported by experimental data showing that the suppression of negative emotions leads to increased food intake in both healthy normal weight students and obese individuals with and without binge eating disorder (BED; Evers et al., 2010; Svaldi et al., 2014). Importantly, negative emotions and their maladaptive regulation are considered key contributing factors to anorexia nervosa (AN; Harrison et al., 2009; Davies et al., 2012), bulimia nervosa (BN; Southward et al., 2013; Lavender et al., 2014), and BED (Vanderlinden et al., 2004; Brockmeyer et al., 2014).

Notably, there is a lack of data on the relevance of emotion regulation difficulties to the group of patients diagnosed as Eating Disorder Not Otherwise Specified/Other Specified Eating or Feeding Disorders (EDNOS/OSFED, as characterized in the 4th and 5th editions of the Diagnostic and Statistical Manual of Mental Disorders, respectively; American Psychiatric Association, 2000, 2013). Because this category includes patients who do not meet full criteria for AN, BN, or BED, individuals included in this category may be more functional, showing subthreshold, or less severe forms of ED. Nonetheless, studies using the DSM-IV criteria highlighted the clinical severity of patients diagnosed as EDNOS, demonstrating that individuals who receive this diagnosis do not differ from those with full ED diagnoses in eating pathology, clinical severity, or general psychopathology (Thomas et al., 2009). Likewise, research suggests that individuals with EDNOS have levels of alexithymia (Nowakowski et al., 2013) and depression (Schmidt et al., 2008) similar to or even higher than those meeting full ED criteria. Thus, although the criteria for ED in general have changed and OSFED differs from EDNOS, evidence suggests emotion regulation difficulties may be just as relevant to OSFED as to the full syndrome ED.

ED have also been related to specific personality traits, including high harm avoidance and low self-directedness for all ED diagnostic subtypes, high novelty seeking for BED and BN, and high reward dependence and persistence for AN (Bulik et al., 1998; Krug et al., 2011; Agüera et al., 2012; Atiye et al., 2015). Notably, more dysfunctional personality traits have been found to predict not only higher ED severity, general psychopathology, and self-harm behaviors, but also worse therapy response and prognosis (Abbate-Daga et al., 2011; Claes et al., 2012; Hintsanen et al., 2012; Rodríguez-Cano et al., 2014).

Research suggests that one pathway through which these personality traits may relate to ED is emotion regulation difficulties. For example, evidence suggests that high levels of neuroticism, behavioral inhibition, and harm avoidance, together with low levels of extraversion, are related to self-report, physiological, and neurological indices of emotion regulation difficulties (Kokkonen and Pulkkinen, 2001; John and Gross, 2004; Ng and Diener, 2009; Di Simplicio et al., 2012; Pickett et al., 2012; Baeken et al., 2014). Likewise, some of these traits (e.g., behavioral inhibition, avoidant personality traits) have been found to be associated with behavioral indices of emotion regulation difficulties, including the unwillingness to experience distress (Tull et al., 2010; Gratz et al., 2013). This association is also evident on a neurobiological level, as high harm avoidance is related to stronger resting state activation and white matter microstructural organization of brain networks associated with emotion regulation (Taddei et al., 2012; Baeken et al., 2014), and correlates with amygdala activation to emotional images (Most et al., 2006; Baeken et al., 2009; Van Schuerbeek et al., 2014). Furthermore, both high levels of harm avoidance and low levels of self-directedness are associated with lower μ-opioidergic neurotransmission in emotion-related brain regions (which is involved in the modulation of emotional reactions; Tuominen et al., 2012). Overall, this research suggests that certain personality traits may increase the risk for emotion regulation difficulties, which, in turn, may increase the risk for various forms of psychopathology, including ED.

Although basic research points to a relation between personality and emotion regulation, there is limited research on the relations between personality and emotion regulation in specific psychiatric disorders. Moreover, most research on the interrelations of personality and emotional functioning in ED has focused on emotional responding vs. emotion regulation *per se*. For example, one such study (Brownstone et al., 2013) found that the relation between affect lability and over-exercising in BN is moderated by compulsive personality traits. Additionally, there is research on the mediating role of anger in the relation between certain personality traits and ED (Krug et al., 2008; Amianto et al., 2012). Research on the interrelations of personality traits and emotions in ED notwithstanding, a growing body of research emphasizes the importance of considering responses to emotions (i.e., emotion regulation) rather than the nature or quality of emotions *per se* when examining psychopathology, both in general (e.g., Gratz and Tull, 2010; Gratz et al., 2013) and with regard to ED in particular (Fox and Power, 2009; Evers et al., 2010). This is a particularly understudied area within the ED literature. One frequently used questionnaire to measure emotion regulation is the Difficulties in Emotion Regulation Scale (DERS; Gratz and Roemer, 2004), which is based on the multidimensional conceptualization of emotion regulation as maladaptive ways of responding to emotions, including a lack of awareness, understanding, and acceptance of emotions, difficulties controlling impulsive behaviors and engaging in goal-directed behaviors when experiencing negative emotions, and a lack of access to effective strategies for modulating emotions. Although, the original version of this measure has been shown to have good reliability and adequate validity in both adults and adolescents (Gratz and Tull, 2010), the Spanish translation of this measure has not yet been validated in the Spanish adult population.

The aims of this study were twofold. Aim 1 was to provide data on the factor structure and validity of the Spanish version of the DERS within a combined clinical-nonclinical sample of Spanish adults with ED and healthy controls (HC). To this end, we examined the factor structure of the DERS within this sample, tested its capacity to discriminate between ED patients and HC, and examined its relations to ED severity, personality traits, and general psychopathology. Aim 2 was to explore the role of emotion regulation difficulties in ED by examining differences in emotion regulation difficulties across ED subtypes (with special attention to OSFED) and exploring the mediating role of emotion regulation difficulties in the relation between key personality traits and ED severity. Based on the existing ED literature, we hypothesized that ED patients would report higher levels of emotion regulation difficulties than HC. We also hypothesized that difficulties in emotion regulation would be associated with higher levels of dysfunctional personality traits, ED severity, and general psychopathology. Finally, consistent with past research indicating an association between certain personality traits (i.e., high harm-avoidance and low self-directedness) and emotion regulation difficulties, we hypothesized that the relation between these personality traits and ED symptoms would be mediated by difficulties in emotion regulation.

## Methods

### Participants

The current study was conducted between April and November 2014. The HC group consisted of 74 undergraduate volunteer students of the University of Barcelona. Students were approached by their professors after course completion to assess their interest in participating in the study. They received course credit in exchange for their participation. An exclusion criterion for the HC group was a self-reported lifetime diagnosis of any ED (Aim 1). Patients (*n* = 134) were recruited from consecutive referrals to the ED unit of Bellvitge University Hospital (Aims 1 and 2). AN (*n* = 30), BN (*n* = 54), BED (*n* = 20), and OSFED (*n* = 30) patients were diagnosed according to the DSM-IV-TR criteria (American Psychiatric Association, 2000) by means of a semi-structured interview [Structured Clinical Interview for DSM Disorders-I] (First et al., 1996) conducted by experienced psychologists and psychiatrists. These diagnoses were reanalyzed *post-hoc* using the recent DSM-5 criteria (American Psychiatric Association, 2013).

Table 1 provides data on the socio-demographic variables of participants in the study, as well as for their age and Body Mass Index (BMI). Most participants in both groups were single. The mean age of the HC group was 21.1 years (*SD* = 4.5) and their mean BMI was 22.1 kg/m^2^ (*SD* = 3.1). The mean age of the ED group was 28.8 years (*SD* = 10.4) and their mean BMI was 25.0 kg/m^2^ (*SD* = 9.1). Analyses revealed statistically significant differences between the HC and ED groups in all socio-demographic variables presented in Table 1. As for differences in these variables across the ED subtypes, results revealed no significant between-group differences for sex, marital status, educational level, or employment status. However, the mean age of participants was higher for the BED group than the other groups, and the mean BMI was higher for the BED group and lower for the AN group, relative to all other ED subtypes.

**Table 1:** **Demographic and selected clinical data for the sample**.

		**HC (*n* = 74)**		**ED sample (*n* = 134)**		***p***	**AN (*n* = 30)**		**BN (*n* = 54)**		**BED (*n* = 20)**		**OSFED (*n* = 30)**		***p***
Sex; *n-%*	Female	51	68.9%	121	90.3%	<0.001	25	83.3%	50	92.6%	16	80.0%	30	100%	0.054
	Male	23	31.1%	13	9.7%		5	16.7%	4	7.4%	4	20.0%	0	0%	
Marital status; *n-%*	Single	72	97.3%	98	73.1%	<0.001	23	76.7%	40	74.1%	10	50.0%	25	83.3%	0.076
	Married	0	0.0%	28	20.9%		4	13.3%	12	22.2%	9	45.0%	3	10.0%	
	Divorced	2	2.7%	8	6.0%		3	10.0%	2	3.7%	1	5.0%	2	6.7%	
Educational level^a^; *n-%*	Primary	0	0%	54	40.3%	<0.001	7	23.3%	27	50.0%	5	25.0%	15	50.0%	0.078
	Secondary	74	100%	51	38.1%		14	46.7%	16	29.6%	12	60.0%	9	30.0%	
	University	0	0%	29	21.6%		9	30.0%	11	20.4%	3	15.0%	6	20.0%	
Employment; *n-%*	Employed	38	51.4%	96	71.6%	0.003	20	67.7%	38	70.4%	6	30.0%	6	20.0%	0.690
Age (years-old)	Mean-SD	21.10	4.47	28.76	10.43	<0.001	28.20	11.21	27.65	8.96	36.65	10.86	26.07	9.82	0.002
BMI (kg/m^2^)	Mean-SD	22.07	3.81	24.95	9.13	0.010	16.84	1.85	25.27	5.96	39.54	10.33	22.80	4.56	<0.001

*AN, anorexia nervosa; BED, binge eating disorder; BMI, Body Mass Index; BN, bulimia nervosa; ED, eating disorder; HC, healthy control; OSFED: other specified eating and feeding disorders; SD, standard deviation*.

a *Primary educational level, no qualification/first qualification age 16; Secondary educational level, qualification for admission to university, age 16–19; University, degree or higher degree*.

### Measures

#### Difficulties in emotion regulation scale (DERS; Gratz and Roemer, 2004)

The DERS is a 36-item self-report measure that assesses individuals' typical levels of emotion dysregulation across six domains: non-acceptance of emotional responses; difficulties pursuing goal-directed behaviors when experiencing negative emotions; difficulties controlling impulsive behaviors when experiencing negative emotions; lack of emotional awareness; limited access to emotion regulation strategies; and lack of emotional clarity. Higher values indicate greater difficulties in emotion regulation. The DERS has been found to demonstrate good reliability (Cronbach's α = 0.93; test–retest reliability over a period ranging from 4 to 8 weeks = 0.88) and adequate construct and predictive validity and is significantly associated with objective (i.e., behavioral, physiological, and neurological) measures of emotion regulation (Gratz and Roemer, 2004; Gratz et al., 2006, 2007; Vasilev et al., 2009; Gratz and Tull, 2010; Goodman et al., 2014). A Spanish version of the DERS was previously validated in the Spanish general adolescent population (Gómez-Simón et al., 2014), and found to have satisfactory fit of the 36 item and six factor model. Internal consistency in this sample was adequate (α between 0.71 and 0.84) with the exception of the Awareness subscale (α = 62).

#### Eating disorders inventory-2 (EDI-2; Garner et al., 1983)

The EDI-2 is a 91-item self-report questionnaire that assesses characteristics of AN and BN on the dimensions of drive for thinness, bulimia, body dissatisfaction, ineffectiveness, perfectionism, interpersonal distrust, interoceptive awareness, maturity fears, asceticism, impulse regulation, and social insecurity. This scale has been validated in a Spanish population (Garner, 1998), obtaining a mean internal consistency of α = 0.63. Internal consistency in the current sample ranged from moderate (ascetic scale, α = 0.70) to excellent (total scale, α = 0.96).

#### Symptom check-list 90 revised (SCL-90-R; Derogatis, 1994)

The SCL-90-R is a 90-item self-report questionnaire measuring psychological distress and psychopathology. The items load on nine symptom dimensions: somatization, obsessive-compulsive, interpersonal sensitivity, depression, anxiety, hostility, phobic anxiety, paranoid ideation, and psychoticism. The global score (Global Severity Index, GSI), is a widely used index of psychopathological distress. The SCL has been validated in a Spanish population obtaining a mean internal consistency of α = 0.75 (Derogatis, 2002). Internal consistency in this sample was between good (paranoid ideation scale, α = 0.83) and excellent (global indexes, α = 0.98).

#### Temperament and character inventory revised (TCI-R; Cloninger, 1994)

The TCI-R is a 240-item self-report questionnaire measuring personality on four temperament and three character dimensions. The temperament dimensions include harm avoidance (e.g., inhibited/passive vs. energetic/outgoing), novelty seeking (e.g., reward-seeking/impulsive vs. uninquiring/reflective), reward dependence (e.g., sociable/socially dependent vs. tough-minded/socially insensitive), and persistence (e.g., perseverant/ambitious vs. inactive/erratic). The character dimensions assess self-directedness (e.g., responsible/goal-directed vs. insecure/inept), cooperativeness (e.g., helpful/empathic vs. hostile/aggressive), and self-transcendence (e.g., imaginative/unconventional vs. controlling/materialistic). The original questionnaire and the Spanish version of the revised questionnaire were validated and showed good psychometric properties (Cloninger, 1994; Gutiérrez-Zotes et al., 2004). Internal consistency in the current sample was good (novelty seeking scale, α = 0.83) to excellent (harm avoidance scale, α = 0.93).

### Procedure

All participants provided written informed consent; the study was conducted according to the Declaration of Helsinki and was approved by the local ethical committee. Patients were evaluated and diagnosed at the ED Unit of the University Hospital of Bellvitge by experienced psychologists and psychiatrists during two assessment sessions. The first assessment session consisted of a face-to-face interview that provided information about current ED symptoms and antecedents, as well as other psychopathological data of interest. The second assessment session involved weight/eating monitoring and the completion of self-report measures (see Section Measures). HC participants were assessed in one session, during which they had their weight and height recorded and completed the relevant self-report measures, as well as a written survey on socio-demographic data, core symptoms of ED, history of psychopathology, and family psychopathology.

### Data analyses

Statistical analyses were carried out with SPSS20 and Stata13 for Windows. The analysis plan included multiple statistical comparisons. In order to control for Type I error inflation, and because the classical Bonferroni's correction method has been criticized for being too conservative, an improved modified procedure was used here: the Holm–Bonferroni method (see, e.g., Gratz and Roemer, 2004; Weinberg and Klonsky, 2009; Cooper et al., 2014). This method has the advantage of being more powerful and especially useful when several highly-correlated test statistics are involved. Additionally, because *p*-values are strongly dependent on sample sizes, we included effect sizes for all comparisons, including partial eta^2^ for ANCOVA models and Cohen's d coefficient for mean differences. Mean differences were considered medium for |*d*| > 0.5 and large for |*d*| > 0.8. Correlation coefficients were considered medium for |*r*| > 0.30 and large for |*r*| > 0.50.

#### Aim 1

First, an Exploratory Factor Analysis (EFA, with Varimax-rotation) and a Confirmatory Factor Analysis (CFA, selecting the Maximum Likelihood and the Robust estimation method) were conducted to examine the internal structure of the Spanish version of the DERS. For the EFA analyses, sample adequacy was based on the Kaiser–Meyer–Olkin measure (for ease of interpretation, 0.90 is considered excellent, 0.80 is considered good, 0.70 is considered moderate, 0.60 is considered low, 0.50 is considered poor, and below 0.50 is considered unacceptable), and Bartlett's test of sphericity was used to test the hypothesis that the correlation matrix is an identity matrix (*p* < 0.05 is indicative that the data set included correlations appropriate for the factor analysis). For the CFA analysis, goodness-of-fit was assessed using the Standardized Root Mean Squared Residuals [(SRMR, adequate fit was considered for SRMR limited to 0.10 (Hu and Bentler, 1999; Kline, 2010)], as well as the Root Mean Squared Error of Approximation (RMSEA), Comparative Fit Index (CFI), and Tucker–Lewis Index (TLI).

Second, analyses of covariance (ANCOVAs), controlling for participants' age, sex, and education level, were conducted to explore the discriminative capacity of the DERS scores in differentiating between controls and ED patients. Third, Pearson-correlation coefficients were conducted to assess the linear associations between DERS scores and the measures of ED (EDI-2 scales), general psychopathology (SCL-90-R), and personality traits (TCI-R dimensions).

#### Aim 2

ANCOVAs, controlling for participants' age, sex, and education level, were conducted to examine group differences in DERS scores between ED subtypes. Structural Equation Modeling (SEM) was used to examine the hypothesized mediating role of emotion regulation difficulties in the relation between personality traits and ED severity. Robust standard errors were estimated and overall goodness-of-fit was assessed through the RMSEA, CFI, TLI, and SMSR. Adequate fit was considered for RMSE < 0.08, CFI > 0.90, TLI > 0.90 and SRMR < 0.10. Global predictive utility of the model was estimated with the Coefficient of Determination (CD).

## Results

### Preliminary analyses

Data was screened for outliers in the DERS scale, with an exclusion criterion of a deviation of more than 3 SD from the sample mean. No outliers were detected. The DERS scores were normally distributed, as shown by non-significant Shapiro–Wilk normality tests (*p* > 0.05 for all subgroups and subscales).

Preliminary analysis to identify possible covariates indicated that the demographic factors sex, age, and education were each significantly related to some of the dependent variables (DERS, TCI-R, and EDI-2) in the whole sample and partly also in the ED sample. Therefore, these variables were controlled for in the following analyses to ensure that any observed associations between the variables of interest are not due to their shared associations with these demographic variables. Nevertheless, since in the ED sample the demographic variables were not significantly related to all of the dependent variables, we run each analyses of Aim 2 without covariates. For the path model, standardized coefficients obtained in the model not adjusted by age and sex were quite similar to those obtained in the adjusted model, but goodness-of-fit was clearly poorer (RMSEA and TLI did not achieve the threshold for adequate fitting, see Figure S1). For the ANOVAS, evidence remained similar wherefore we report the adjusted results for all analyses. Unadjusted results can be consulted in the Supplementary Material (Tables S2, S3 and Figure S1).

### Aim 1

#### Internal structure of the DERS: factor analyses

Table S1 shows the factor loadings obtained in the EFA for the one-dimensional factor solution and the six-factor solution, as well as the standardized coefficients of the CFA for the Spanish version of the DERS for the whole sample. Prior to the factor analyses, inverse items (1, 2, 6, 7, 8, 10, 17, 20, 22, 24, 34) were reversed so that higher scores indicate greater emotion regulation difficulties. Sample adequacy for the EFA was excellent (Kaiser–Meyer–Olkin = 0.927, Bartlett's test *p* < 0.001). The one-dimension factor solution in the EFA was acceptable, providing a factor with high loadings for all the DERS items, excellent internal consistency (Cronbach's alpha, α = 0.96), and a moderate percentage of explained variance (41.1%). The six-factor solution was also acceptable and corresponded closely to the original six-factor solution identified in Gratz and Roemer (2004) paper (with a few exceptions involving high cross-loadings of items on the Strategies and Clarity factors with other factors; i.e., items 1, 9, 22, 28, 30, 31, and 36). All factors in the six-factor solution had good internal consistency (α coefficients ranging from 0.81 for the Impulse factor and 0.92 for the Non-acceptance factor), and accounted for 64.0% of the cumulative explained variance. Additionally, all factors were significantly correlated with one another (with correlations between factors ranging from *r* = 0.49 to 0.77), with the exception of the Awareness factor (see Table S1 for intercorrelations between the factors).

Results of the CFA support the acceptability of the 6-factor solution; the standardized coefficients for all DERS items on their respective (and theorized) dimension were significant and moderate to high in magnitude. Additionally, the 6-factor solution demonstrated adequate goodness of fit across all indices (RMSEA = 0.076, CFI = 0.903, TLI = 0.900, SRMR = 0.088), supporting the adequacy of this factor solution.

The following analyses were performed using the DERS raw scores (obtained as the direct sum of the items) for the six original subscales and the DERS total scale.

#### Discriminative capacity of the DERS

Table 2 shows the results of the ANCOVAs comparing the mean DERS scores for the HC and ED groups, adjusted for participants' sex, age, and educational level. As expected, participants in the ED group reported greater difficulties in emotion regulation across all dimensions; all effect sizes accompanying these mean differences were medium to large. These results provide support for the discriminative capacity of the DERS in differentiating between HC and ED cases. Furthermore, we explored the sensibility of the DERS to discriminate each specific ED subgroup from HC (see Supporting Information, Table S4). The Goals subscale was the only one which couldn't successfully discriminate HC from two ED subgroups, namely AN and BED. All the other subscales had good discriminative capacity for each ED diagnostic subtype.

**Table 2:** **Discriminative capacity of the DERS scores to differentiate between healthy controls and ED patients**.

**DERS-scale**	**HC (*n* = 74)**		**ED (*n* = 134)**		**Means comparison: ANCOVA**				
	**Mean**	***SD***	**Mean**	***SD***	***MD***	***F*-stat**	**^1^*p***	***η*^2^**	**|*d*|**
Non-acceptance of emotional responses	12.3	5.01	18.3	6.84	6.02	31.15	<0.001	0.135	1.00^**^
Difficulties engaging in goal directed behavior	13.8	4.42	16.3	4.95	2.44	8.72	0.004	0.042	0.52^*^
Impulse control difficulties	10.7	3.63	15.9	6.14	5.20	30.56	<0.001	0.133	1.03^**^
Lack of emotional awareness	14.9	4.44	18.3	5.06	3.48	16.89	<0.001	0.078	0.73^*^
Limited access to emotion regulation strategies	15.9	5.88	23.6	8.27	7.65	34.26	<0.001	0.147	1.07^**^
Lack of emotional clarity	10.3	3.91	14.9	5.02	4.56	31.72	<0.001	0.137	1.01^**^
Total score	78.0	18.39	107.3	27.69	29.34	46.54	<0.001	0.190	1.25^**^

*Results of all analyses are adjusted for participant age, sex, and educational level*.

*ED, eating disorder; HC, Healthy Control; MD, mean difference; SD, standard deviation; η^2^, Partial eta2; |d|, Cohen's d*.

*^1^p-values are adjusted for multiple testing using the Holm–Bonferroni method*.

* Medium effect size for d > 0.50 and

** *large effect size for d > 0.80*.

#### Associations of the DERS with ED severity, psychopathology, and personality traits

Table 3 shows the correlations of the DERS scale scores with the clinical measures of ED symptom severity (EDI-2), general psychopathology (SCL-90-R), and personality (TCI-R). In the ED group, all of the DERS scales were significantly positively correlated with the EDI-2 total score and all the SCL-90-R scale scores (such that greater emotion regulation difficulties were associated with higher ED severity and greater psychopathology), with the exception of the DERS Awareness scale (which was not significantly associated with the other clinical measures).

**Table 3:** **Correlations of the DERS scores with the clinical measures for the control and ED samples**.

	**ED sample (*n* = 134)**							**Control sample (*n* = 74)**						
	**N-acc**.	**Goals**	**Impulse**	**Aware**	**Strategies**	**Clarity**	**Total**	**N-acc**.	**Goals**	**Impulse**	**Aware**	**Strategies**	**Clarity**	**Total**
Body mass index	0.09	0.00	−0.03	0.10	0.14	0.11	0.10	−0.06	0.04	−0.06	0.01	−0.01	−0.07	−0.03
EDI: Total score	**0.49**	**0.49**	**0.50**	0.15	**0.59**	**0.55**	**0.62**	–	–	–	–	–	–	–
SCL-90-R: Somatization	**0.33**	**0.35**	**0.35**	0.11	**0.48**	**0.37**	**0.45**	0.30	0.19	0.14	−0.14	0.27	0.17	0.24
SCL-90-R: Obsesse/comp.	**0.45**	**0.52**	**0.43**	0.05	**0.54**	**0.36**	**0.53**	**0.34**	0.27	0.08	−0.03	0.27	**0.37**	**0.33**
SCL-90-R: Interpers. sen.	**0.54**	**0.54**	**0.48**	0.18	**0.69**	**0.53**	**0.66**	**0.30**	0.20	0.13	−0.11	**0.40**	**0.41**	**0.34**
SCL-90-R: Depressive	**0.56**	**0.56**	**0.54**	0.14	**0.69**	**0.50**	**0.68**	**0.39**	0.25	0.26	−0.17	**0.50**	0.24	**0.39**
SCL-90-R: Anxiety	**0.46**	**0.48**	**0.50**	0.10	**0.56**	**0.37**	**0.56**	**0.42**	0.22	0.22	−0.19	**0.38**	0.29	**0.35**
SCL-90-R: Hostility	**0.36**	**0.48**	**0.53**	0.16	**0.55**	**0.38**	**0.55**	**0.35**	0.16	**0.33**	−0.05	0.22	**0.32**	**0.33**
SCL-90-R: Phobic anxiety	**0.40**	**0.42**	**0.35**	0.13	**0.51**	**0.31**	**0.48**	**0.30**	0.22	0.13	−0.20	0.30	0.16	0.24
SCL-90-R: Paranoid	**0.46**	**0.48**	**0.44**	0.13	**0.58**	**0.45**	**0.57**	0.17	0.11	0.15	0.01	0.30	0.25	0.25
SCL-90-R: Psychotic	**0.49**	**0.44**	**0.48**	0.10	**0.52**	**0.42**	**0.55**	**0.38**	0.16	0.19	−0.05	**0.36**	**0.42**	**0.37**
SCL-90-R: GSI score	**0.53**	**0.55**	**0.53**	0.13	**0.67**	**0.48**	**0.65**	**0.41**	0.24	0.22	−0.12	**0.43**	**0.34**	**0.39**
SCL-90-R: PST score	**0.50**	**0.48**	**0.47**	0.18	**0.60**	**0.46**	**0.60**	**0.42**	0.26	0.27	−0.02	**0.44**	**0.45**	**0.46**
SCL-90-R: PSDI score	**0.50**	**0.48**	**0.46**	0.06	**0.59**	**0.41**	**0.57**	**0.31**	0.20	0.11	−0.22	**0.33**	0.13	0.23
TCI-R: Novelty seeking	−0.07	0.03	0.06	−0.03	−0.02	0.03	−0.01	−0.05	0.05	0.09	−0.01	−0.03	0.17	0.04
TCI-R: Harm avoidance	**0.37**	**0.43**	**0.32**	0.10	**0.51**	**0.41**	**0.48**	0.29	0.28	0.06	0.04	**0.47**	0.25	**0.37**
TCI-R: Reward depen.	−0.09	−0.05	−0.09	−0.28	−0.04	−0.21	−0.15	0.02	−0.10	−0.10	**−0.41**	−0.08	**−0.37**	−0.24
TCI-R: Persistence	−0.16	**−0.33**	−0.26	−0.12	**−0.37**	−0.23	**−0.33**	0.05	−0.04	0.08	**−0.37**	−0.08	−0.29	−0.16
TCI-R: Self-directedness	**−0.42**	**−0.46**	**−0.46**	−0.20	**−0.54**	**−0.54**	**−0.59**	−0.18	−0.14	−0.18	−0.09	**−0.39**	**−0.47**	**−0.37**
TCI-R: Cooperativeness	−0.08	−0.16	−0.16	−0.17	−0.17	−0.24	−0.21	0.01	−0.14	−0.23	−0.22	−0.11	−0.24	−0.22
TCI-R: Self-Transc.	0.09	0.02	0.01	−0.18	−0.04	−0.11	−0.04	0.18	−0.01	0.17	−0.14	0.02	0.11	0.08

*Bold, Medium to large effect size for correlation |r|> 0.30; –, Not available for this group*.

All DERS scales (except Awareness) were also significantly correlated with the personality traits of harm avoidance (positive association) and self-directedness (negative association). The DERS Goals, Strategies, and Total scale scores were also negatively correlated with persistence. BMI was not significantly correlated with any of the DERS scales in either the ED or HC groups (see Table 3). Moreover, BMI was not significantly correlated with the DERS total score in any of the ED subtypes. However, within the BED subgroup, BMI was significantly correlated with the Goals (*r* = −0.31) and Awareness (*r* = 0.39) scales of the DERS.

**Figure 1:**
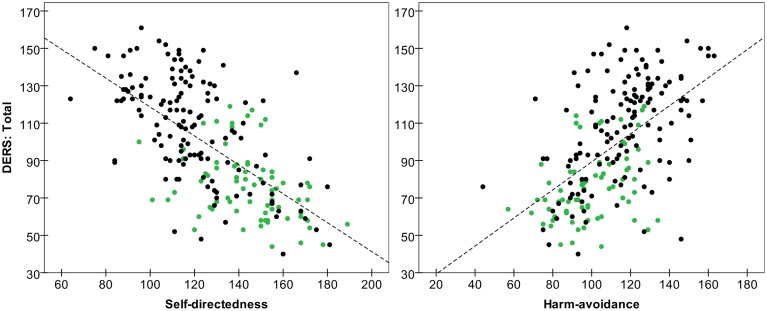
**Scatter-plot of the DERS total score with TCI-R self-directedness and harm avoidance scales**. ED patients are in black and HC participants are in green. Dashed line represents total line fit.

The pattern of associations within the HC group was somehow different. The DERS scales demonstrating the most robust associations with the clinical measures were as follows: (a) the Non-acceptance scale correlated significantly with most SCL-90-R scales (excluding somatization and paranoid); (b) the total DERS score and the Strategies and Clarity subscales correlated with many SCL-90-R scores; (c) the Awareness scale correlated negatively with the TCI-R reward dependence and persistence scales; (d) the DERS total score and the Strategies subscale correlated positively with TCI-R harm avoidance and negatively with TCI-R self-directedness; and (e) the Clarity scale correlated negatively with the TCI-R reward dependence and self-directedness scales. Figure 1 contains the scatter-plots of the distribution of DERS total score with TCI-R self-directedness and harm avoidance scales.

### Aim 2

#### Differences in emotion regulation difficulties across ED subtypes

Results of an ANCOVA (controlling for sex, age, and educational level) comparing mean differences in emotion regulation difficulties across the four ED subtypes (AN, BN, BED, and OSFED) revealed significant differences between ED subtypes for the DERS total scale and the Strategies, Goals and Non-acceptance subscales (*t*-values and effect sizes see Table 4, *p* < 0.05). Scores on the Awareness, Clarity, and Impulse subscales did not differ between groups (*p*s > 0.10). The AN group reported lower mean scores than the BN group on the Non-acceptance, Goals and Strategies subscales. The AN group also reported lower mean scores than the OSFED group on the Goals and Strategies subscales. Table 4 presents both the mean DERS-scores for the ED subtypes (AN, BN, BED, and OSFED) and the pairwise comparisons between the ED subtypes.

**Table 4:** **Comparison of the DERS scores between different ED subtypes**.

	**Adjusted means and standard deviations**								**ANOVA adjusted by age, education and sex**				
	**AN (*n* = 30)**		**BN (*n* = 54)**		**BED (*n* = 20)**		**OSFED (*n* = 30)**		**Significant pairwise comparisons**				
	Mean	SD	Mean	SD	Mean	SD	Mean	SD	Contrast	MD	t	*p*	|d|
Non acceptance of emotional responses	16.39	7.52	19.61	6.00	17.99	6.46	19.36	7.59	AN-BN	−3.22	2.01	0.047	0.50^*^
Difficulties engaging in goal directed behavior	14.43	4.81	17.23	5.11	15.64	3.80	17.30	4.99	AN-BN	−2.80	2.43	0.017	0.56^*^
									AN-OSFED	−2.87	2.16	0.032	0.59^*^
Impulse control difficulties	14.22	5.58	16.57	5.49	15.62	6.64	17.06	7.18	−	−	−	−	−
Lack of emotional awareness	18.07	5.43	17.99	4.86	19.82	5.46	17.63	4.95	−	−	−	−	−
Limited access to emotion regulation strategies	20.00	7.52	25.37	7.91	24.21	7.52	24.46	9.14	AN-BN	−5.37	2.79	0.006	0.70^*^
									AN-OSFED	−4.46	2.01	0.046	0.53^*^
Lack of emotional clarity	14.41	5.45	14.96	4.86	15.16	4.69	15.40	5.19	−	−	−	−	−
Total score	97.52	30.08	111.74	24.91	108.45	23.19	111.20	30.73	AN-BN	−14.22	2.19	0.030	0.51^*^

MD, mean difference; SD, standard deviation; |d|, Cohen's d; AN, anorexia; BN, bulimia nervosa; BED, binge eating disorder; OSFED, other specified eating and feeding disorders.

* *Medium (d > 0.50) and large (d > 0.80) effect sizes*.

#### Path analysis of the interrelations between personality traits, emotion regulation difficulties, and ED

**Figure 2:**
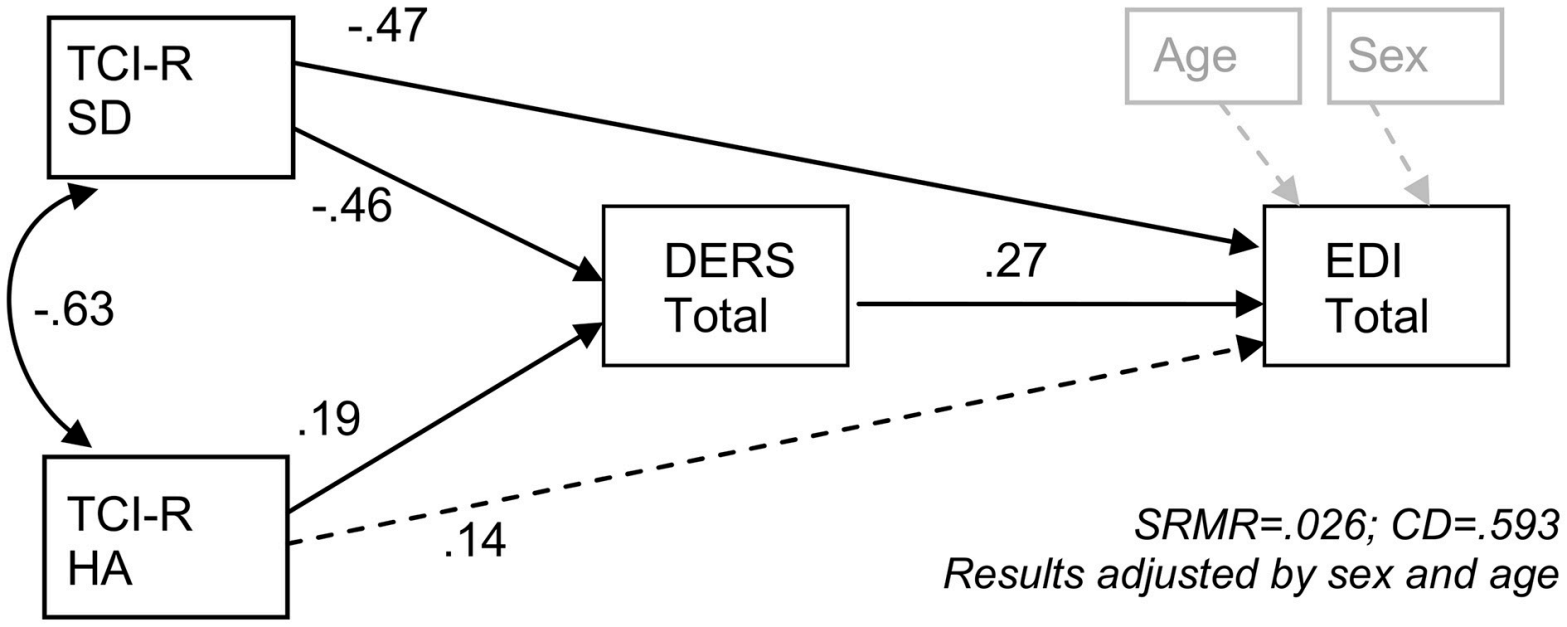
**SEM of the proposed mediation model of emotion regulation difficulties mediating the relation of personality traits and eating disorder severity**. CD, Coefficient of Determination; DERS, Difficulties in Emotion Regulation Scale; EDI, Eating Disorders Inventory; HA, Harm Avoidance; SD, Self-Directedness; SRMR, Standardized Root Mean Squared Residuals; TCI-R, Temperament and Character Inventory—Revised.

Figure 2 contains the results of the path analysis of the theorized associations among the personality traits (self-directedness and harm-avoidance), emotion regulation difficulties (DERS total score), and ED severity (EDI-2-total score), adjusted for patient sex and age. Including these factors as covariates allows us to examine the interrelations of personality, emotion regulation, and ED symptoms when accounting for their shared associations with sex and age. This model shows that the DERS total score mediates the relation between the personality traits and ED severity; specifically, low scores on the self-directedness scale and high scores on the harm avoidance scale predicted higher scores on the DERS total scale, and high scores on the DERS total scale were associated with higher EDI-2 total scores. In addition, self-directedness had a direct effect on EDI-2 total scores (such that lower levels of this personality trait were related to higher scores on the EDI-2 total scale). Adequate goodness-of-fit of the path model was demonstrated across all fit indices (RMSEA = 0.061, CFI = 0.994, TLI = 0.975 and SRMR = 0.026), and the global predictive capacity was high (CD = 0.59).

## Discussion

The aims of the present study were to examine the factor structure and validity of the Spanish version of the DERS in a combined clinical and nonclinical sample of Spanish adults, and to examine the role of emotion regulation difficulties in ED by examining differences in emotion regulation difficulties across ED subtypes (with special attention to OSFED) and exploring the mediating role of emotion regulation difficulties in the relation between key personality traits and ED severity.

The factor structure of the DERS in our adult Spanish sample was comparable to that obtained in the original study, with support provided for the original six-factor solution. Furthermore, consistent with previous studies with Anglo–Saxon samples (Harrison et al., 2009; Brockmeyer et al., 2014; Lavender et al., 2014), the DERS and its subscales demonstrated good internal consistency and convergent validity. Results also support the discriminative capacity of the DERS, as the DERS total score and each subscale differentiated between the HC and ED patients, with ED patients reporting significantly greater difficulties in emotion regulation than the HC group (consistent with past research using the DERS in ED samples; Harrison et al., 2009; Brockmeyer et al., 2014; Lavender et al., 2014).

Greater difficulties in emotion regulation were associated with higher self-reported general psychopathology in both the HC and ED groups, although the associations were smaller in the HC group. Furthermore, although BMI was not significantly associated with emotion regulation difficulties in either the ED or HC groups, greater difficulties in emotion regulation were associated with greater ED severity among the ED patients. Indeed, with the exception of difficulties in emotional awareness, all DERS subscales were significantly correlated with ED severity. Positive correlations between measures of emotion regulation difficulties and ED severity have been found in former studies (Gupta et al., 2008; Svaldi et al., 2012; Gianini et al., 2014; Lavender et al., 2014). Likewise, the absence of a significant association between difficulties in emotional awareness (as assessed with the DERS Awareness subscale) and ED was found previously in BN patients (Lavender et al., 2014). One possible explanation for the lack of significant relations between the Awareness subscale and ED pathology may be that being aware of one's emotions is a necessary but not sufficient condition for adaptive emotion regulation, with emotional awareness alone not automatically resulting in more adequate emotion regulation and fewer psychiatric difficulties. Nevertheless, another study found a small association between deficits in emotional awareness and ED symptoms (Svaldi et al., 2012). Further research is needed to draw conclusions about the exact relations among awareness of emotions, adequate emotion regulation, and psychopathology.

Our hypothesis that certain personality traits would be associated with emotion regulation difficulties was also supported by our findings. Greater difficulties in emotion regulation in the ED group were related to more dysfunctional personality traits. More specifically, high harm avoidance, low self-directedness, and low persistence were associated with greater difficulties engaging in goal-directed behavior when experiencing negative emotions and with less access to adaptive emotion regulation strategies. Moreover, higher levels of harm avoidance and lower levels of self-directedness were related to greater non-acceptance of emotions, greater difficulties controlling impulses when experiencing negative emotions, and lower emotional clarity. Lack of emotional awareness was not associated with harm avoidance or self-directedness in the ED sample. Thus, the personality traits reflected in self-directedness (i.e., poor resourcefulness, helplessness, irresponsibility) and harm avoidance (i.e., behaviorally and socially inhibited, fear of uncertainty, easily tired) are associated with difficulties in all dimensions of emotion regulation assessed by the DERS, with the exception of emotional awareness.

Although the DERS has been used to assess emotion regulation difficulties among ED patients in past studies, this is the first study to our knowledge to include the category of OSFED. Within our sample, the greatest difficulties in emotion regulation were found in the BN and the OSFED groups, which did not differ from one another. Additionally, the AN group reported lower difficulties in emotion regulation than both the BN and OSFED groups. Findings that the OSFED patients in our sample reported levels of emotion regulation difficulties comparable to those reported by the BN patients and greater than those reported by the AN patients provide preliminary support for the relevance of emotion regulation difficulties to this particular ED subtype. These findings are consistent with past research demonstrating that patients diagnosed as EDNOS do not differ from those with full ED diagnoses in clinical severity or overall psychopathology (Thomas et al., 2009), and provide further evidence for the clinical relevance of the OSFED subtype. Conversely, findings that the AN patients reported lower levels of emotion regulation difficulties than the BN or OSFED groups differ from previous findings suggesting that AN patients report levels of emotion regulation difficulties comparable to BN patients, with BED patients reporting fewer problems with emotion regulation (Svaldi et al., 2012; Brockmeyer et al., 2014). Given that the AN patients in this sample reported levels of emotion regulation difficulties comparable to those previously reported by patients with AN (Harrison et al., 2010; Brockmeyer et al., 2012), findings of lower levels of emotion regulation difficulties among the AN (vs. BN and OSFED) patients in this sample may reflect the greater severity of the BN and OSFED groups in our study (relative to past studies), rather than a lack of emotion regulation difficulties in the AN group. Specifically, differences in the sampling or recruitment methods across studies in this area may influence the nature of the ED sample. Alternatively, these findings may reflect cultural differences in the clinical presentation and severity of ED.

Results of the SEM suggest that the relation of the personality traits of harm avoidance and self-directedness to ED severity is mediated by difficulties in emotion regulation. More specifically, results suggest that whereas self-directedness influences ED severity through both direct and indirect pathways, harm avoidance may only have an indirect effect on ED severity through difficulties in emotion regulation. Although the cross-sectional nature of our study precludes conclusions about the precise nature and direction of the observed relationships, there is sufficient evidence to support our proposed model. For example, twin studies suggest that a substantial proportion (~30–40%) of the variance in temperament and personality traits is explained by genetic components (Heath et al., 1994; Ando et al., 2002; Gillespie et al., 2003; Garcia et al., 2013) and longitudinal studies suggest that personality predicts emotion regulation (Kokkonen and Pulkkinen, 2001; Xia et al., 2014). Furthermore, research suggests that maladaptive emotion regulation predicts psychopathology rather than the other way around (McLaughlin et al., 2011; Bardeen et al., 2013; Berking et al., 2014; Goodwin et al., 2014; Wirtz et al., 2014).

Thus, research suggests that a biologically-based and heritable temperament predisposes the development of more or less adaptive emotion regulation strategies. Notably, although Cloninger's (1993) original model of personality distinguished between temperament and character (with the former referring to genetically determined traits, such as harm avoidance, and the latter corresponding to environmentally-determined traits that are thought to emerge over time, such as self-directedness), evidence suggests that the hypothesis of a neurobiological distinction between temperament and character cannot be upheld (Farmer and Goldberg, 2008) and provides support for a genetic basis of character traits as well (Ando et al., 2002; Gillespie et al., 2003; Garcia et al., 2013). Thus, both harm avoidance and self-directedness traits—through biological mechanisms such as hypothalamic–pituitary–adrenal axis and autonomic nervous system functioning—may play a central role in how an individual will respond to and manage environmental influences. Resiliency to psychopathology has been suggested to be associated with the development of a stable emotion regulation network (Cisler et al., 2013), whereas the absence of adaptive emotion regulation can result in various psychopathological symptoms (Aldao et al., 2010). With regard to ED in particular, the biopsychosocial model of ED proposes that biologically- and genetically-based vulnerabilities (which are seen in personality traits) influence reactivity to external stimuli, integration of childhood experiences, storage of emotional schemas, and the acquirement (or lack thereof) of adaptive emotion regulation strategies (Kochanska et al., 2009; Calkins et al., 2013; Shapero and Steinberg, 2013), with the lack of adaptive regulation strategies resulting in the use of maladaptive strategies, such as starvation, binge eating, or self-injurious behavior, to avoid or escape unwanted or overwhelming negative emotions (Fox and Power, 2009; Haynos and Fruzzetti, 2011; Ivanova et al., 2015; Lavender et al., 2015).

There are some limitations of this study that warrant mention. First, the cross-sectional nature of this study precludes conclusions about the direction or temporal ordering of the observed relationships, or the development and interrelations of the constructs of interest over time. Second, the sample size was relatively small, particularly for some of the specific ED subgroups. Thus, results of the within-ED group comparisons need to be interpreted with caution. In addition, the patient groups were too small to subdivide the AN group into binge-purging and restrictive subtypes, which may influence results. Third, our sample did not include a sufficient number of male participants to examine gender effects or the moderating role of gender in the observed relations. Fourth, we relied exclusively on the DERS to assess emotion regulation difficulties. Although the DERS is based on a multidimensional conceptualization of emotion regulation difficulties (Gratz and Roemer, 2004) and, thus, assesses several distinct dimensions of emotion regulation difficulties, it is not exhaustive and other dimensions of emotion regulation difficulties (including emotion regulation strategy use and emotional avoidance) remain unexamined. Thus, future studies would benefit from the use of multiple self-report measures of emotion regulation difficulties (in addition to objective behavioral and/or physiological measures of emotion regulation) in order to examine emotion regulation deficits in ED and their mediating role in the personality-ED relation.

Finally, longitudinal studies beginning earlier in the lifespan are needed to fully understand the influence of inherited temperament on the acquisition of emotion regulation strategies and the development of adaptive emotion regulation. There is preliminary evidence to suggest that harm avoidance is an endophenotype (Gottesman et al., 2003) for psychological illness (Choe et al., 2013; Markett et al., 2013; Calati et al., 2014), whereas difficulties in emotion regulation are learned responses to emotions that may be attributed to environmental influences to a greater extent (Kanakam et al., 2013). However, the precise nature of the relation between harm avoidance and emotion regulation remains unclear. Further research in this area would also be helpful in developing new intervention strategies for ED patients. Training in emotion regulation may be one way to influence temperament and character phenotypes and improve treatment outcomes (Bulik et al., 1998; Fagundo et al., 2013; Kaye et al., 2015). Cognitive behavioral therapy teaches patients to identify and label thoughts and feelings, as well as to decrease avoidance behavior and promote more adaptive behavioral choices. As we have seen in a previous study (Agüera et al., 2012), these learned strategies during treatment help to change personality traits, and, as a result, may promote the development of more adaptive emotion regulation strategies. Treatments focused specifically on promoting more adaptive emotion regulation, such as emotion regulation group therapy (Gratz et al., 2014, 2015) could lead to even better results and target more directly and efficiently both emotion regulation difficulties and the psychopathology stemming from those difficulties.

### Conflict of interest statement

The authors declare that the research was conducted in the absence of any commercial or financial relationships that could be construed as a potential conflict of interest.

## Abbreviations

AN: Anorexia Nervosa
ANCOVA: Analysis of Covariance
ANOVA: Analysis of Variance
BED: Binge Eating Disorder
BMI: Body Mass Index
BN: Bulimia Nervosa
CD: Coefficient of Determination
CFA: Confirmatory Factor Analysis
CFI: Comparative Fit Index
DERS: Difficulties in Emotion Regulation Scale
ED: Eating Disorder
EDI-2: Eating Disorders Inventory
EDNOS: Eating Disorder Not Otherwise Specified
EFA: Exploratory Factor Analysis
HC: Healthy Control
OSFED: Other Specified Eating or Feeding Disorders
RMSEA: Root Mean Squared Error of Approximation
SCL-90-R: Symptom Check List
SEM: Structural Equation Modeling
SD: Standard Deviation
SRMR: Standardized Root Mean Squared Residuals
TCI-R: Temperament and Character Inventory
TLI: Tucker-Lewis Index.
