# Supplementary material for: Emotion regulation in disordered eating: Psychometric properties of the Difficulties in Emotion Regulation Scale among Spanish adults and its interrelations with personality and clinical severity
Source: Front Psychol. 2015 Jun 30;6:907. doi: 10.3389/fpsyg.2015.00907 (PMC4485313; doi:10.3389/fpsyg.2015.00907)
Supplement: Supplementary file 1 [file DataSheet1.DOCX]

***Supplementary Material***

**Emotion regulation in disordered eating: Psychometric properties of the Difficulties in Emotion Regulation Scale among Spanish adults and its interrelations with personality and clinical severity**

**Authors**

Ines Wolz^1,2,3^, Zaida Agüera^1,2^; Roser Granero^2,4^; Susana Jiménez-Murcia^1,2,3^, Kim L. Gratz^5^, José Manuel Menchón^1,3,6^ & Fernando Fernández-Aranda^1,2,3*^

**Affiliations**

^1^Department of Psychiatry, University Hospital of Bellvitge-IDIBELL, Barcelona, Spain.

^2^Ciber Fisiopatologia Obesidad y Nutrición (CIBERObn), Instituto Salud Carlos III, Barcelona, Spain

^3^Department of Clinical Sciences, School of Medicine, University of Barcelona, Spain

^4^Department of Psychobiology and Methodology. University Autònoma of Barcelona, Spain.

^5^Department of Psychiatry and Human Behavior, University of Mississippi Medical Center, Jackson, Mississippi, USA.

^6^Ciber Salud Mental (CIBERSAM), Instituto Salud Carlos III, Barcelona, Spain

***Address for correspondence:** Fernando Fernández-Aranda, Ph.D., FAED, Department of Psychiatry and CIBEROBN, University Hospital of Bellvitge, c/ Feixa Llarga s/n, 08907-Barcelona, Spain (e-mail: ffernandez@bellvitgehospital.cat; Tel. +34-932607227)

1. **Supplementary Figures and Tables**

## Supplementary Tables

**Table S1.** Results of the exploratory factor analysis (EFA) and the confirmatory factor analysis (CFA) for the Spanish version of the DERS (total sample, n=208).

|  |  | EFA | EFA: rotated factor matrix | | | | | | CFA | |
| --- | --- | --- | --- | --- | --- | --- | --- | --- | --- | --- |
|  |  | F1 | F1 | F2 | F3 | F4 | F5 | F6 | Coef. | Scale |
| 11 | Angry with myself for feeling that way | **.739** | **.797** | .239 | .073 | .182 | .106 | -.130 | .805 | Non-  acceptance |
| 12 | Embarrassed for feeling that way | **.716** | **.714** | .075 | .100 | .267 | .189 | .082 | .772 |  |
| 21 | Ashamed with myself for feeling that way | **.796** | **.772** | .084 | .148 | .283 | .142 | .151 | .840 |  |
| 23 | I feel like I am weak | **.713** | **.562** | .398 | .158 | .116 | .280 | .289 | .733 |  |
| 25 | I feel guilty for feeling that way | **.771** | **.758** | .349 | .068 | .119 | .122 | .136 | .862 |  |
| 29 | Irritated with myself for feeling that way | **.790** | **.742** | .264 | .136 | .170 | .152 | .187 | .857 |  |
| 13 | I have difficulty getting work done | **.671** | .281 | **.679** | -.042 | .211 | .015 | .121 | .737 | Goals |
| 18 | I have difficulty focusing on other things | **.768** | .241 | **.768** | -.053 | .238 | .100 | .132 | .879 |  |
| 20 | I can still get things done | **.534** | .001 | **.454** | .313 | .209 | .102 | -.085 | .425 |  |
| 26 | I have difficulty concentrating | **.761** | .292 | **.766** | .024 | .185 | .199 | .011 | .852 |  |
| 33 | I have difficulty thinking about anything else | **.733** | .159 | **.696** | .002 | .271 | .249 | .174 | .833 |  |
| 3 | I experience emotions out of control | **.466** | .098 | .133 | -.005 | **.409** | .296 | .065 | .474 | Impulse |
| 14 | I become out of control | **.823** | .371 | .350 | .114 | **.680** | .125 | .133 | .876 |  |
| 19 | I feel out of control | **.844** | .342 | .376 | .112 | **.739** | .162 | .132 | .849 |  |
| 24 | I can remain in control of my behaviour | **.556** | .177 | .212 | .414 | **.513** | .122 | .077 | .631 |  |
| 27 | I have difficulty controlling my behaviours | **.754** | .335 | .395 | .048 | **.609** | .224 | .164 | .848 |  |
| 32 | I lose control over my behaviours | **.751** | .278 | .321 | .160 | **.723** | .168 | .101 | .863 |  |
| 2 | I pay attention to how I feel | **.749** | .082 | .055 | **.801** | .104 | .182 | .104 | .853 | Awareness |
| 6 | I am attentive to my feelings | **.685** | .172 | -.040 | **.771** | .072 | .001 | .060 | .824 |  |
| 8 | I care about what I am feeling | **.660** | .113 | -.095 | **.777** | -.038 | .025 | .061 | .782 |  |
| 10 | I acknowledge my emotions | **.557** | .005 | .065 | **.654** | .108 | .232 | .006 | .607 |  |
| 17 | My feelings are valid and important | **.531** | -.197 | -.065 | **.438** | -.197 | -.003 | -.382 | .307 |  |
| 34 | I take time to figure out what I’m feeling | **.368** | .083 | .101 | **.416** | .053 | .159 | -.214 | .429 |  |
| 15 | I will remain that way for a long time | **.779** | .373 | .448 | .088 | .290 | .172 | **.458** | .801 | Strategies |
| 16 | I’ll end up feeling very depressed | **.750** | .396 | .409 | .121 | .299 | .253 | **.425** | .817 |  |
| 22 | I know that I can find a way to feel better | **.543** | .054 | **.325** | .497 | .197 | .051 | **.224** | .517 |  |
| 28 | I believe nothing make myself feel better | **.719** | .284 | **.389** | .285 | .433 | .220 | **.342** | .799 |  |
| 30 | I start to feel very bad about myself | **.829** | **.581** | .346 | .196 | .305 | .262 | **.352** | .863 |  |
| 31 | I believe that wallowing in it is all I can do | **.526** | .273 | **.409** | .178 | .282 | .166 | **.291** | .678 |  |
| 35 | It takes me a long time to feel better | **.726** | .247 | .407 | .118 | .246 | .214 | **.583** | .738 |  |
| 36 | My emotions feel overwhelming | **.748** | .323 | **.481** | .143 | .434 | .219 | **.295** | .805 |  |
| 1 | I am clear about my feelings | **.705** | .143 | .129 | **.571** | .182 | **.467** | .099 | .732 | Clarity |
| 4 | I have no idea how I am feeling | **.764** | .242 | .168 | .301 | .209 | **.706** | .079 | .850 |  |
| 5 | Difficulties making sense out of my feelings | **.790** | .251 | .279 | .329 | .198 | **.709** | .077 | .881 |  |
| 7 | I know exactly how I am feeling | **.660** | .192 | .044 | **.529** | .166 | **.489** | .082 | .728 |  |
| 9 | I am confused about how I feel | **.691** | .252 | .233 | .225 | .232 | **.696** | .108 | .815 |  |
|  | Cronbach's alpha (α) | .956 | .922 | .864 | .898 | .807 | .916 | .901 | SRMR=.088 | |
|  | Correlation between factors | F2 | .550 |  |  |  |  |  |  |  |
|  |  | F3 | .631 | .676 |  |  |  |  |  |  |
|  |  | F4 | .189 | .127 | .236 |  |  |  |  |  |
|  |  | F5 | .713 | .743 | .775 | .264 |  |  |  |  |
|  |  | F6 | .557 | .492 | .606 | .532 | .653 |  |  |  |

**Table S2**. Discriminative capacity of the DERS-scores to differentiate between healthy controls and ED patients without controlling for socio-demographic variables age, sex and education.

|  | Control  (*n=*74) | | ED  (*n*=134) | | Means comparison:  T-TEST | | | |
| --- | --- | --- | --- | --- | --- | --- | --- | --- |
| DERS-scale | Mean | SD | Mean | SD | MD | t-stat | ^1^p | \|d\| |
| Non-acceptance of emotional responses | 11.81 | 5.01 | 18.69 | 6.84 | 6.88 | 7.55 | <0.001 | 1.15** |
| Difficulties engaging in goal directed behavior | 13.59 | 4.42 | 16.43 | 4.95 | 2.84 | 4.09 | <0.001 | 0.60* |
| Impulse control difficulties | 10.53 | 3.63 | 16.03 | 6.14 | 5.50 | 7.00 | <0.001 | 1.09** |
| Lack of emotional awareness | 15.10 | 4.44 | 18.16 | 5.06 | 3.07 | 4.35 | <0.001 | 0.64* |
| Limited access to emotion regulation strategies | 15.48 | 5.88 | 23.83 | 8.27 | 8.35 | 7.63 | <0.001 | 1.16** |
| Lack of emotional clarity | 10.12 | 3.91 | 14.95 | 5.02 | 4.82 | 7.12 | <0.001 | 1.07** |
| Total score | 76.63 | 18.4 | 108.1 | 27.7 | 31.45 | 8.71 | <.001 | 1.34** |

Note. ED = eating disorder; HC = Healthy Control; MD = mean difference; SD = standard deviation; |d| = Cohen’s d.

^1^p-values include Holm-correction for multiple statistical tests.

*Medium effect size for d>0.50 and **large effect size for d>0.80.

**Table S3**. Comparison of the DERS-scores between ED subtypes without controlling for socio-demographic variables sex, age and education.

|  | AN; *n*=30 | | BN; *n*=54 | | BED; *n*=20 | | OSFED; *n*=30 | | ANOVA and significant pairwise comparison | | | | | |
| --- | --- | --- | --- | --- | --- | --- | --- | --- | --- | --- | --- | --- | --- | --- |
|  | Mean | SD | Mean | SD | Mean | SD | Mean | SD | F | p | Contrast | MD | *p* | \|d\| |
| Non-accept. | 16.60 | 7.52 | 19.67 | 6.00 | 17.90 | 6.46 | 19.53 | 7.59 | 1.56 | .202 | AN<BN | 3.07 | .050 | 0.45 |
| Goals | 14.53 | 4.81 | 17.30 | 5.11 | 15.40 | 3.80 | 17.43 | 4.99 | 2.84 | .041 | AN<BN | 2.76 | .014 | 0.56* |
|  |  |  |  |  |  |  |  |  |  |  | AN<OSFED | 2.90 | .022 | 0.59* |
| Impulse | 14.13 | 5.58 | 16.63 | 5.49 | 15.35 | 6.64 | 17.30 | 7.18 | 1.66 | .179 | AN<OSFED | 3.17 | .046 | 0.50* |
| Aware | 17.87 | 5.43 | 18.11 | 4.86 | 19.20 | 5.46 | 17.87 | 4.95 | 0.34 | .793 | --- | --- | --- | --- |
| Strategy | 19.87 | 7.52 | 25.50 | 7.91 | 23.75 | 7.52 | 24.83 | 9.14 | 3.34 | .021 | AN<BN | 5.63 | .003 | 0.73* |
|  |  |  |  |  |  |  |  |  |  |  | AN<OSFED | 4.97 | .018 | 0.59* |
| Clarity | 14.07 | 5.45 | 15.07 | 4.86 | 14.80 | 4.69 | 15.70 | 5.19 | 0.54 | .653 | --- | --- | --- | --- |
| Total | 97.07 | 30.08 | 112.3 | 24.91 | 106.4 | 23.19 | 112.7 | 30.72 | 2.64 | .074 | AN<BN | 15.21 | .016 | 0.55* |
|  |  |  |  |  |  |  |  |  |  |  | AN<OSFED | 15.60 | .028 | 0.51* |

Note. AN = anorexia nervosa; BED = binge eating disorder; BN = bulimia nervosa; HC = healthy controls; MD = mean difference; OSFED = other specified eating and feeding disorders; SD = standard deviation;|d| = Cohen’s d. *Medium (d>0.50) and large (d>0.80) effect sizes.

**Table S4.** Discriminative capacity of the DERS scores for the different ED subtypes: ANOVA adjusted by sex, age and studies levels.

|  | Descriptives: adjusted means and SD | | | | | | | | | | Factor | | Pairwise comparisons. HC = reference group | | | | | | | |
| --- | --- | --- | --- | --- | --- | --- | --- | --- | --- | --- | --- | --- | --- | --- | --- | --- | --- | --- | --- | --- |
|  | HC; *n=74* | | AN; *n=30* | | BN; *n=54* | | BED; *n=20* | | OSF.; *n=30* | | Group | | AN vs HC | | BN vs HC | | BED vs HC | | OSF. vs HC | |
|  | Mean | SD | Mean | SD | Mean | SD | Mean | SD | Mean | SD | F | ^1^p | MD | *\|d\|* | MD | *\|d\|* | MD | *\|d\|* | MD | *\|d\|* |
| Non-acceptance | 12.2 | 5.0 | 16.2 | 7.5 | 19.3 | 6.0 | 18.0 | 6.5 | 19.0 | 7.6 | 9.09 | <.001 | **3.99** | **0.62*** | **7.11** | **1.29*** | **5.78** | **1.00*** | **6.77** | **1.05*** |
| Goals | 13.7 | 4.4 | 14.4 | 4.8 | 17.1 | 5.1 | 15.6 | 3.8 | 17.1 | 5.0 | 3.93 | .004 | 0.68 | 0.15 | **3.36** | **0.70*** | 1.85 | 0.45 | **3.35** | **0.71*** |
| Impulse | 10.6 | 3.6 | 14.1 | 5.6 | 16.5 | 5.5 | 15.6 | 6.6 | 17.0 | 7.2 | 8.99 | <.001 | **3.42** | **0.73*** | **5.87** | **1.26*** | **4.92** | **0.92*** | **6.41** | **1.13*** |
| Aware | 14.8 | 4.4 | 18.2 | 5.4 | 18.2 | 4.9 | 19.9 | 5.5 | 17.8 | 4.9 | 4.77 | .001 | **3.43** | **0.69*** | **3.34** | **0.72*** | **5.11** | **1.03*** | **2.99** | **0.64*** |
| Strategy | 15.7 | 5.9 | 19.8 | 7.5 | 25.2 | 7.9 | 24.2 | 7.5 | 24.3 | 9.1 | 11.40 | <.001 | **4.08** | **0.60*** | **9.48** | **1.36*** | **8.44** | **1.25*** | **8.57** | **1.12*** |
| Clarity | 10.3 | 3.9 | 14.3 | 5.4 | 14.9 | 4.9 | 15.2 | 4.7 | 15.3 | 5.2 | 8.04 | <.001 | **4.01** | **0.85*** | **4.59** | **1.04*** | **4.90** | **1.14*** | **5.04** | **1.10*** |
| Total score | 77.5 | 18.4 | 97.1 | 30.1 | 111.2 | 24.9 | 108.5 | 23.2 | 110.6 | 30.7 | 13.51 | <.001 | **19.6** | **0.79*** | **33.8** | **1.54*** | **31.0** | **1.48*** | **33.1** | **1.31*** |

^1^p-values include Holm-correction for multiple statistical tests. Bold: significant pairwise comparison. *Moderate (d>0.50) and large (d>0.80) effect size.

. |d|: Cohen’s d.

AN = anorexia nervosa; BN = bulimia nervosa; BED = binge eating disorder; HC = healthy controls; MD: mean difference; OSF. = other specified eating and feeding disorders; SD = standard deviation.

## Supplementary Figures

##
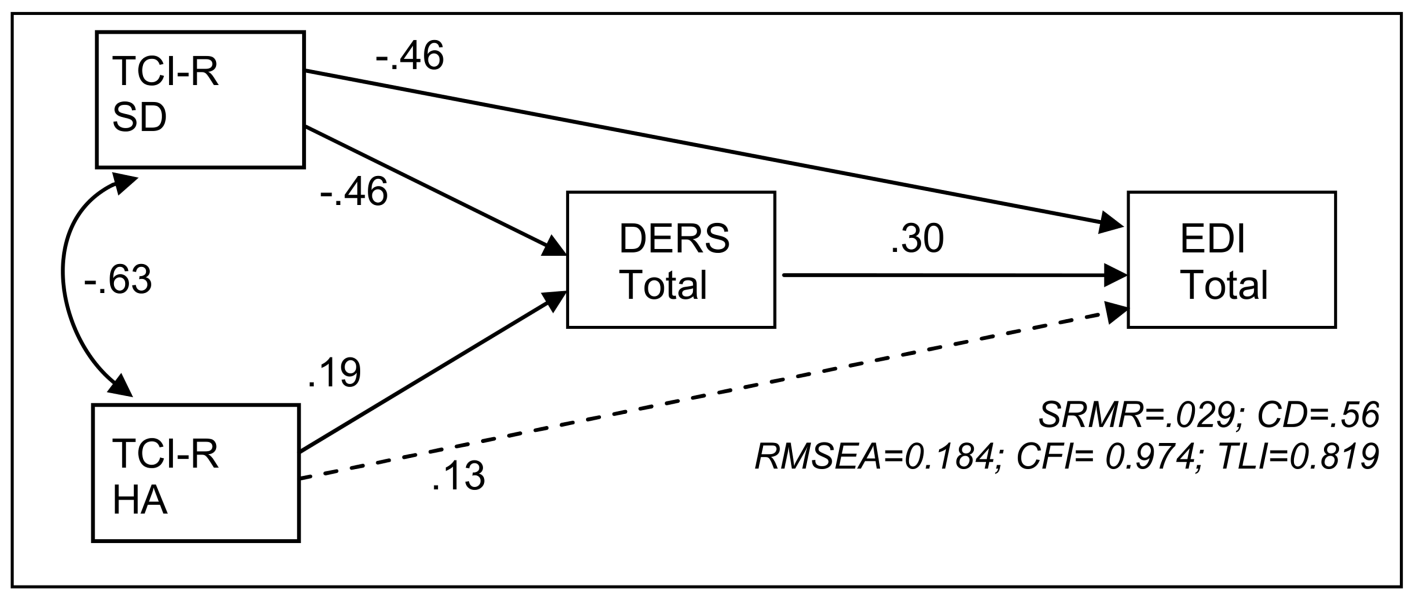


## Figure S1. SEM of the proposed mediation model of emotion regulation difficulties mediating the relation of personality traits and eating disorder severity, without controlling for participants’ age and sex.
